# Impact of Pricing and Product Information on Consumer Buying Behavior With Customer Satisfaction in a Mediating Role

**DOI:** 10.3389/fpsyg.2021.720151

**Published:** 2021-12-13

**Authors:** Huiliang Zhao, Xuemei Yao, Zhenghong Liu, Qin Yang

**Affiliations:** ^1^Department of Product Design, School of Fine Arts, Guizhou Minzu University, Guiyang, China; ^2^School of Mechanical Engineering, Guizhou University, Guiyang, China; ^3^School of Data Science and Information Engineering, Guizhou Minzu University, Guiyang, China; ^4^School of Mechanical Engineering, Guiyang University, Guiyang, China

**Keywords:** product pricing, product packaging, consumer buying behavior, consumer satisfaction, confirmatory factor analysis, structural equation modeling

## Abstract

The relationship between product pricing and product packaging plays an important role in the buying behavior of consumers, whereas customer satisfaction plays a mediating role. To test these hypotheses, research was conducted on university students in China. Questionnaire-based convenience sampling was conducted on 500 students for data collection using online and offline sources. A total of 367 (73%) students responded, and 17 questionnaires were rejected due to missing information. SPSS and AMOS software were used for the data analysis. Product pricing and product information were independent variables in this study, whereas consumer buying behavior was a dependent variable. Customer satisfaction is mediated by one dependent and two independent variables. Confirmatory factor analysis, path analysis, and discriminant validity in structural equation modeling revealed that product pricing and packaging had a statistically significant relationship with the buyer decision process. The introduction of satisfaction as a mediating variable led to the observation of full mediation in the case of product pricing and partial mediation in product packaging. Given the results of this research, product managers should adopt pricing tactics along with product packaging to influence the buying intentions of consumers.

## Introduction

In the competitive market of commodities, products, varieties, consumers, ethnicities, and preferences, product pricing and product packaging information descriptions have a considerable influence on the buying behavior of consumers. To explore the cumulative effects of product pricing and packaging on the buying behavior of consumers of different ethnicities, it is essential to research these aspects of marketing. It is worth mentioning that consumer satisfaction also plays a decisive and mediating role in the development and molding of buying behavior of consumers (Larsen et al., 2017). It is believed that pricing has a significant effect on the buying behavior of consumers because the higher a product is priced, the fewer units are sold. By contrast, products selling at prices lower than the market rate are assumed to sell at a higher volume (Sadiq M. W. et al., 2020). Several studies have shown that pricing is more critical and relevant to consumer buying behavior (Huo et al., 2021).

When discussing the combined effect of product pricing and packaging relationships on consumer buying behavior, pricing alone plays a more critical role than packaging, which has a partial role in buying behavior (Jabarzare and Rasti-Barzoki, 2020). Thus, using this analogy, products can be sold, surprisingly, at a much higher volume. One can increase the prices of the products if the competitor products are scarce in the market or if the manufacturers are low in number. This behavior may not affect the number of sales or the attitude of the consumer toward buying. If the product is already in abundance in the market, then pricing will definitely play an important role because the increase in price will discourage customers from buying it. Similarly, if prices are lowered under such market conditions, then consumers will increase the amount that they purchase significantly.

Even though product pricing has a greater influence than product packaging on the decision process of a buyer (Pratama and Suprapto, 2017; Abdullah et al., 2021), high prices in a highly competitive market can lose customers permanently due to the effect of increased pricing (Kotler et al., 2012). While talking about the packaging of products, it should be kept in mind that packaging has a significant relationship on consumers and their decision making about product purchases (Sadiq M. W. et al., 2020). For example, quality, color, and material can have a positive effect on consumers (Rambabu and Porika, 2020). Most consumers desire a range of product choices when purchasing, in terms of packaging. Thus, marketers should place a premium on creative and exclusive packaging that is distinctive in scale, instruction, convenience, product design, and form when compared with rivals in the market segment (Li et al., 2021). Marking a product with accurate information adds to its value. Consumers are attracted to detailed labels, content, and packaging. Many people are influenced by the way a product is packaged and presented in the market. While the product itself may be of any quality, the relationship it produces through its packaging has a strong influence on the purchasing attitude of the consumer. Nowadays, eco-friendly packaging is essential. Thus, advertisers should prioritize this factor and employ best practices to the maximum degree possible, including eco-friendly recyclable packaging (Abdullah et al., 2021). Consumer buying behavior also has a lot to do with product selling and buying (Brun et al., 2014), although some customers are not influenced by the packaging or labeling of products, buying is demand-driven or need-oriented by most consumers.

However, super packaging or labeling of products may not attract the consumer for several reasons. One of the primary reasons may be the high price and packaging, announcing the excellent quality of the product. In such cases, there may be a lack of interest by the consumer toward attractive packaging; instead, they may prefer to buy local products that are cheap and readily available in the market. According to Tu and Chih (2013), consumer satisfaction is another aspect of product selling and consumer buying behavior. It also plays a mediating role in product buying behavior, pricing, and packaging (Rambabu and Porika, 2020). Even though a price might be negotiable and the product is provided with helpful information and good, decent packaging, there is a lot to do to satisfy a consumer. All of these factors are correlated with consumer satisfaction. If the consumer is satisfied with all these, they may buy the product, but there is no guarantee of this. Thus, consumer buying behavior is also influenced by satisfaction (Brun et al., 2014). This study seeks to answer several questions to explain consumer buying behavior in relation to product pricing and packaging, with consumer satisfaction as a mediating factor. In this work, we first present a brief review of this research, which differs from the current literature in various respects. The research has generated several findings.

• Product prices significantly correlate with consumer buying behavior.
• The product information available on packaging influences the consumer’s buying behavior.
• Satisfaction plays a mediating role in consumer buying behavior.
• Pricing of the product plays an essential role in customer satisfaction.
• Product information available on labels plays a significant role in customer satisfaction.

The remainder of this work is structured as follows: Section “Review of Literature and Hypothesis Development” presents a review of previous studies supporting different theoretical frameworks. Section “Research Methodology” presents the methodology adopted for the empirical analysis. Section “Data Analysis and Results” presents the results of this analysis. Section “Conclusion and Recommendations” concludes the present study, limitations and future directions.

## Review of Literature and Hypothesis Development

### Product Pricing and Consumer Buying Behavior

Product pricing seems to be the only direct element that generates revenue and indicates the success or failure of a product or service. As a result, the researchers in this study chose to emphasize this aspect. Manali (2015) carried out research into the theoretical dimensions of consumer purchasing behavior and the factors that affect it. He analyzed the relationship between consumer buying behavior and factors affecting the buying process and decisions of the consumers. His research provides enough evidence to show that the internal and external influences of a consumer have a major relationship with their purchasing behavior.

According to Al-Salamin et al. (2015), good prices of well-known brands negatively affect the purchasing process. Young people are eager to buy brands, but their low income hinders them from doing so. The only aspect of the marketing mix that generates revenue is price, whereas the others generate costs. The authors also noted that the purchasing decisions of consumers focus on their price perception and what they think about the actual price of a product. The main goal of marketing is to understand how customers move toward their price perception. We are all customers, no matter how old, educated, wealthy, or talented. Understanding customer behavior thus becomes a critical challenge for advertisers, distributors, and salespeople. Therefore, we hypothesized the following:

H_1_: Product pricing is significantly correlated with consumer buying behavior.

### Product Packaging and Consumer Buying Behavior

Packaging a product with relevant product details contributes positively to consumer buying behavior. Names, features, and product packaging attract consumers. Many people are influenced by the packaging and marketing of items. While a product may be of any quality, the impact on customer purchasing is essential (Rundh, 2009; Li et al., 2021; Naseem et al., 2021). The aim of this study was to determine the effect of product pricing and information about product packaging on the buying behavior of consumers. Innovation in product labeling and packing often has a major relationship with demand, which is why there are many methods for this type of action plan if a company wants to pursue this strategy with regard to its product packaging. When it comes to packaging, many buyers want a range of product choices. Therefore, marketers should pay high prices for innovative and exclusive packaging that differentiate their products from the competition in terms of size, guidance, functionality, product innovation, and shape (Rundh, 2009; Li et al., 2021; Sarfraz et al., 2021). For the target consumer, product packaging acts as an outstanding networking tool, ultimately increasing their awareness levels. Packaging must highlight key aspects of the product and brand, such as material composition, purpose, and quality. To show respect for customers, packaging should include all of this information in regional languages. Not only is efficient packaging important for storing and preserving products, but it is also important for creating an interest in and generating actions toward purchasing the product. Packaging that is environmentally friendly has become increasingly important. As a result, marketers should place a high priority on this aspect and use best practices to the greatest possible extent, including the use of environmentally friendly recycled materials (Deliza and MacFie, 2001; Abdullah et al., 2021; Mohsin et al., 2021).

H_2_: Product information on packaging is significantly related to consumer purchasing behavior.

### Satisfaction of Consumers and Their Buying Behavior

Customer value and customer satisfaction are considered important parameters for the relationship between customer value and the willingness to sacrifice (Zechmeister et al., 1997). This sacrifice is made in accordance with an exchange mechanism that includes transaction costs and the risk of the goods of the company. According to Larsen et al. (2017), customers will be disappointed in the future if the ratio value considered by the economic sacrifice of customers with the goods sold by the company does not meet their expectations. Customers will be satisfied if the ratio value is sufficient or exceeds their expectations. Another analysis of consumer value examines the understanding of customers of the quality and benefits of toothpaste in relation to price sacrifice. Social, emotional, and functional values are all aspects of customer value (Keller and Kotler, 2012).

Customer satisfaction is evaluated by obtaining feedback from customers after purchasing products or services, and then comparing it with their expectations. Customer satisfaction is calculated using the performance requirements of products or services that are capable of satisfying the needs and desires of customers. A satisfied consumer is a consumer who believes that the products or services were worth purchasing, which would encourage them to buy the products again. On the other hand, a frustrated consumer will persuade other consumers not to buy the same brand, which ultimately causes switching to rival brands. According to Tu and Chih (2013), “customer satisfaction is perceived as affecting repurchasing intentions and actions, which, in turn, contributes to an organization’s potential sales and income.”

H_3_: Satisfaction plays a mediating role in consumer buying behavior.

### Role of Product Pricing on Consumer Satisfaction

Price is regarded as something that can be calculated according to several measures, such as a reasonable price, a competitive price, a discounted price, a retailer’s price, and price suitability. Value is a higher-level definition than quality and price because it is more individualistic and personal. A satisfied consumer believes that the value of goods and services is comparable with the price, which will encourage them to repurchase the products. According to Zeithaml (1988), “quality can be characterized as superiority or excellence in a broad sense.” From the customer’s perspective, “The price is given up or sacrificed to get the product or service” (Zeithaml, 1988). According to Bei and Chiao (2001), “[P]rice is described as giving or sacrificing for the acquisition of a service or product,” while Kotler et al. (2012) proposed that “the price is the amount paid for a product or service and the sum of the value exchanged by consumers for the advantages of a product or service available or being used.” The perceptions of customers of a given price can have a direct relationship with the their decision to buy a product (Zechmeister et al., 1997). Customers will pay attention to the prices paid by their peers, and no one wants to spend more money than their peers do. The fairness of a price can affect the perception of consumers of the product, and ultimately their desire to become a consumer.

H_4_: The pricing of a product plays a significant role in customer satisfaction.

### Role of Product Packaging on Consumer Satisfaction

Packaging and labeling can be considered one of the most important tools in marketing and communication, which means that a thorough examination of their components and their relationships with consumer buying behavior is necessary. According to Joewono and Kubota (2007), consumer satisfaction results from product and service reviews based on customer perceptions and a broad assessment of the overall consumption experience. It is suggested that customer satisfaction affects repurchase intentions and actions, which, in turn, determine potential sales and revenue for a company. According to Zeithaml (2000), consumer satisfaction is measured on a multidimensional scale that includes service quality, product quality, scenario factors, personal factors, and price factors.

Product packaging plays a variety of roles. It provides information about the product and the company, connects them with customers, and ensures product quality (Naseem et al., 2020; Rambabu and Porika, 2020). It is important to remember that packaging has a significant influence on customers and their purchasing decisions. Consumers react positively to quality, color, and content. Similarly, if a product is labeled with accurate information about the product, it increases the value of the product. Consumers respond to a product’s specific name, ingredients, and packaging. Many consumers are concerned about the way a product is designed and advertised. Although the quality of the product itself may vary, the effect of packaging on customer purchasing decisions is important.

H_5_: Product information available on labels plays a significant role toward customer satisfaction.

### Theoretical Support of the Study

The following research was conducted to investigate underlying issues. This study is a continuation of expectancy disconfirmation theory (EDT) and social cognitive theory (SCT). Both theories provide a strong background for conducting this research. According to EDT, the satisfaction of consumers is linked to the expectation and perception of product quality. A consumer sets an expectation before examining a product in real time. This comparison of preset expectations with real-sense performance is the basis of EDT. In this study, consumer satisfaction plays a mediating role between product pricing, product packaging, and consumer buying behavior. The expectations of consumers are based on the price of the product, information on product packaging, and perceived quality.

**FIGURE 1:**
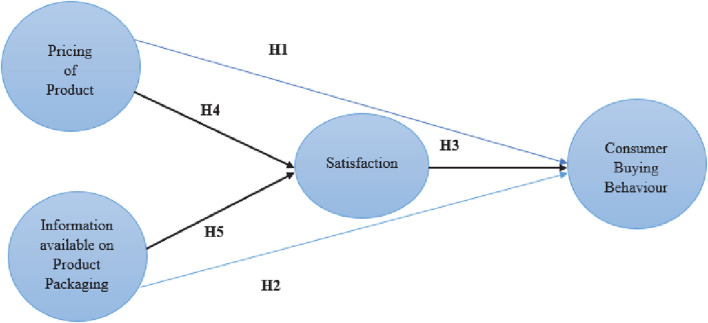
Theoretical framework.

The other central backbone of this research is SCT, developed by Bandura (2012), which explains that learning takes place in a social context with a complex and reciprocal relationship between the individual, their environment, and their actions. The emphasis on social relationships, and also external and internal social reinforcement, is a distinctive feature of SCT. SCT considers the specific ways in which people maintain their behavior and interact with others. It also considers the specific ways in which people learn and sustain behaviors and the social context in which they do so. According to this theory, past experiences strengthen ideas and expectations, all of which affect whether a person maintains his/her attitudes. Many behavioral models that are used in studies related to health do not include behavior maintenance; instead, they focus on behavior initiation. This is a shame because the real purpose of public health is to maintain conduct rather than initiate it. SCT aims to illustrate how people monitor and reinforce their actions to achieve goal-directed behavior that can be managed. Thus, the product pricing and packaging of a product with useful information on labels will surely correlate with consumer buying behavior that will persist. The customer will buy or not buy in the future on the basis of the expectations and perceptions of the product once his behavior about the product has already been initiated. A conceptual framework was developed to focus on the specific variables. The framework consists of the hypotheses shown in Figure 1.

## Research Methodology

The research methodology of a study represents an essential and integral part of the entire process and explains how science contributes to aims. The behavioral approach of respondents, i.e., expectations, evidence, observations, knowledge of reality, and individual point of view, can be summarized by analytical parameters. According to James and Vinnicombe (2002), the assurance of objectivity in the scientific procession is compulsory. Furthermore, a perspective emphasizing social variable is considered essential by the society for practical implications (Blaikie, 2007). Their innovative discoveries and interpretation are leading activities of label research.

### Research Design

In this research, the structure of behavior science by Zechmeister et al. (1997) is followed with mediation and description for the problem-solving process. The main focus of this research is the state of mind, mood swings, variations in feelings, and behavior toward the specific situation of the respondents. In addition, the organizational performance in the market and consumer buying behavior can solve many problems by approaching the cooperative feedback process with peers and accumulating knowledge. The analysis of buying behavior may be categorized as “co-oriented” or “comparative.” According to behavioral science, these two factors have real meaning. This study seeks to understand the effect of product pricing and packaging on the buying behavior of consumers. At the same time, satisfaction plays its role as a mediating variable (Zechmeister et al., 1997; Bollen and Pearl, 2013). For data collection, self-administered questionnaires were used for quantitative analysis.

### Study Population

The sample of this study comprises students from different universities in China. The main reason for choosing university students is that recent research concentrates on product pricing with consumer buying behavior while considering university students as their population. The population selection is based on the area of interest and importance, which covers the objectivity of this research. Divergent online and offline sources were used to collect analytical data. The questionnaires were circulated among 500 students, and the 367 replied to us regarding that, and so the aggregate received response was 73%. Seventeen answers received from respondents were rejected due to incomplete information, and 350 were finalized for the analytical process. This study used convenience sampling for data collection. Bonds-Raacke and Raacke (2012) suggested that field examinations should use a questionnaire. The researcher used a questionnaire to collect the data in this study. SPSS software was used to check the quality, validity, and scale reliability of the instrument.

## Data Analysis and Results

SPSS and AMOS software were used for the data analysis. Table 1 presents the reliability analysis results. Product pricing and product information are independent variables in this study, whereas consumer buying behavior is a dependent variable. In this study, satisfaction is mediated between two independent variables and one dependent variable. All variables have acceptable reliability alpha values.

**TABLE 1:** Reliability analysis.

Variables	Items	Cronbach’s Alpha value
Product pricing	12	0.70
Product packaging	7	0.72
Satisfaction	7	0.76
Consumer buying behavior	7	0.73

Table 2 shows the descriptive statistics. The mean value of product pricing is 3.4, where product information has a mean value of 3.9, satisfaction has a mean value 3.6, and consumer buying behavior has a mean value of 3.8.

**TABLE 2:** Descriptive statistics.

Variables	Mean	Std. deviation	N
Product pricing	3.40	0.96	350
Product packaging	3.90	0.88	350
Customer satisfaction	3.60	0.79	350
Consumer buying behavior	3.80	0.66	350

### Instrument

The product price measuring scale was introduced by Lichtenstein et al. (1993). The Likert scale ranges from strongly agree to strongly disagree, and this scale was used in this research with slight modifications. The Lichtenstein et al. (1993) ranking was further verified by confirmatory factor analysis (CFA) analysis to meet the requirements of this research. The measuring scales of Brun et al. (2014) and Zekiri and Hasani (2015) were used to measure the product packaging and customer satisfaction. The behavior of consumers toward buying decisions, the measurement scale of Bagga and Bhatt (2013) is used with slight modification to fit the scale for scope and broaden the view of this research. All predefined models/scales were rated on 5-point Likert scale, with higher numerical values indicating greater satisfaction.

### Confirmatory Factor Analysis

The pooled CFA is more reliable than other versions and the most up-to-date approach. The AMOS 24 is used to check the relationship among variables (Afthanorhan et al., 2014; Chong et al., 2014).

**FIGURE 2:**
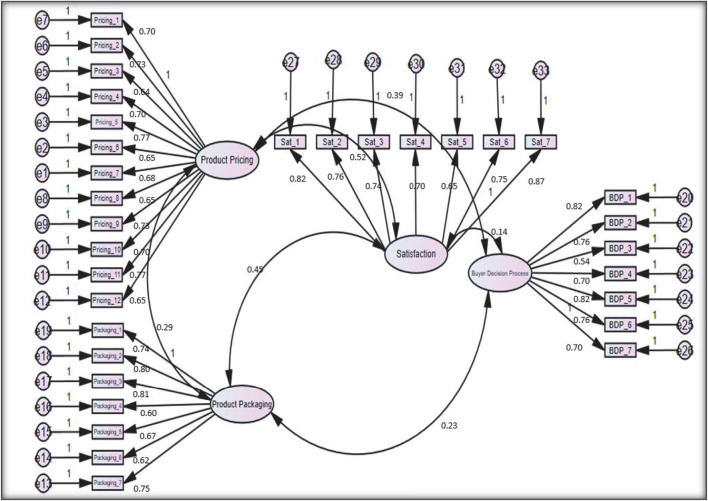
Pooled confirmatory factor analysis.

The results of Table 3 declare the structural fitness of the model by meeting all criterion requirements. The reliability values or factor loading of individual items are presented in Figure 2. The findings of Table 4 have also covered the composite reliability of a wide scale. The composite reliability is indicated by the reliability of the measurement scales while reporting reliability (Netemeyer et al., 2003).

**TABLE 3:** Pooled CFA model fitness tests.

Category name	Index name	Full index name	Analysis value	Acceptable value	Literature
Absolute fit	RMSEA	Root Mean Square of Error Approximation	0.05	< 0.80	Browne, 1993
Incremental fit	CFI	Comparative fit index	0.91	> 0.90	Bentler, 1990
Parsimonious fit	Chisq/df	Chi Square/Degrees of freedom	2.43	< 3	Hu and Bentler, 1999

**TABLE 4:** Factor loading of items.

Scale	Items	Factor loadings	Scale reliability
Product pricing	I am very concerned about low prices, but I am equally concerned about product quality.	0.71	0.70
	When grocery shopping, I compare the prices of different brands to be sure I get the best value for the money.	0.74	
	When purchasing a product, I always try to maximize the quality I get for the money I spend.	0.64	
	When I buy products, I like to be sure that I am getting my money’s worth.	0.70	
	I generally shop around for lower prices on products, but they must still meet certain quality requirements before buying them.	0.78	
	When I shop, I usually compare the “price per ounce” information for brands I normally buy.	0.66	
	I always check prices at the grocery store to be sure I get the best value for the money I spend.	0.69	
	I am not willing to go to extra effort to find lower prices.	0.65	
	I will grocery shop at more than one store to take advantage of low prices.	0.74	
	The money saved by finding low prices is usually not worth the time and effort.	0.70	
	I would never shop at more than one store to find low prices.	0.78	
	The time it takes to find low prices is usually not worth the effort.	0.66	
Product packaging	The packaging color impacts my buying behavior	0.74	0.72
	The label of the package is important for me	0.80	
	The quality of the packaging material is important for me	0.82	
	The package design has an impact on me during my purchase	0.61	
	The printed information on the package helps me to purchase the specific product	0.68	
	The language used on the package influences my buying decision	0.63	
	Innovation and practicality in product packaging is important for me during purchasing	0.76	
Customer satisfaction	I am very satisfied with the ease of use of this product	0.83	0.76
	I am very satisfied with the information provided by this product	0.76	
	I am very satisfied with the personalization offered by this product for me.	0.74	
	My experience with this product is very satisfactory.	0.71	
	I am very satisfied with the packaging of this product.	0.66	
	This product fulfills my needs	0.76	
	The information on product packaging helps me in purchasing this product	0.87	
Consumer buying behavior/Buyer decision process	I usually read online reviews of products before making a purchase decision.	0.83	0.73
	Personal contact and communication with salesperson are important while shopping	0.76	
	Touching or seeing the products in person is an important part of the shopping experience	0.54	
	Blogs are an important source of information regarding products and services	0.71	
	Viral information (videos/articles etc.) influences my perception toward the products	0.83	
	I generally consult family and friends before making a purchase	0.76	
	I usually seek expert opinion online before purchasing a high involvement product	0.71	

### Assessment of Discriminant Validity

Discriminant validity was measured using HTMT analysis by considering two determinants, i.e., supposed to be related or unrelated. The value of cut-off criteria for strict discriminant validity was 0.850, and for liberal discriminant validity it was 0.900 (Henseler et al., 2015), obtained by employing discriminant validity. The following discriminant validity criteria have provided the results of Table 5.

**TABLE 5:** HTMT analysis.

	Product pricing	Product packaging	Satisfaction	Buyer decision process
Product pricing				
Product packaging	0.29			
Satisfaction	0.26	0.20		
Buyer decision process	0.21	0.09	0.04	

### Path Analysis in Structural Equation Modeling

In this study, structural equation modeling was used to determine the proposed relationships. Exogenous variables were included in this analysis to allow for the study of endogenous variables using AMOS 24. Here, we can see whether the independent and dependent variables are linearly related to each other. The analytical observations and their mean values are tabulated and linked with the collected information. The results of Table 6 declare the structural fitness of the model by meeting all criterion requirements.

**TABLE 6:** SEM, model fitness tests.

Category name	Index name	Full index name	Analysis value	Acceptable value	Literature
Absolute fit	RMSEA	Root Mean Square of Error Approximation	0.05	< 0.80	Browne, 1993
Incremental fit	CFI	Comparative fit index	0.72	> 0.90	Bentler, 1990
Parsimonious fit	Chisq/df	Chi Square/Degrees of freedom	1.92	< 3	Hu and Bentler, 1999

**FIGURE 3:**
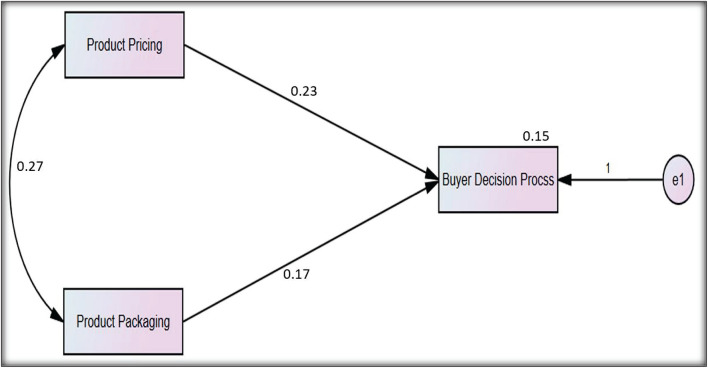
Direct effects of path analysis.

Figure 3 shows the direct effects of the independent variables on the dependent variable. In this figure, the mediator variable is missing from this path analysis diagram to capture the direct correlation of the independent variable on the dependent variable.

**FIGURE 4:**
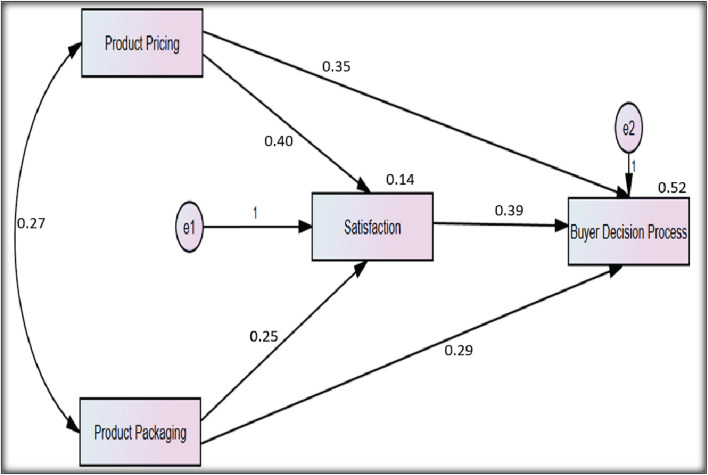
Indirect direct effects of path analysis.

Table 7 shows that H_1_, H_3_, and H_5_ are statistically significant, and their P-value is less than 0.05, which shows the 95% confidence interval. The structural equation modeling with the path analysis is presented in Figure 4. The path analysis declared the nature of variables, i.e., two variables are independent: one is the mediator and the other one is dependent.

**TABLE 7:** Results of indirect effects.

Hypothesis	Causal path	Lower bound	Upper bound	P-value	Standardized estimated
H^1^	Product Pricing → Consumer Buying Behavior	−0.16	0.09	0.00	0.23
H^3^	Product Packaging → Consumer Buying Behavior	−0.18	0.03	0.05	0.17
H^5^	Satisfaction → Consumer Buying Behavior	−0.13	0.04	0.03	0.39

The findings of Table 8 indicate that both hypotheses are statistically significant, but the observed mediation values for these hypotheses differ. H_2_ is statistically significant but has a full mediation effect, whereas H_4_ is statistically significant and has a partial mediation effect.

**TABLE 8:** Results of indirect effects.

S/R	Hypothesis	Direct beta without mediation	Direct beta with mediation	Indirect beta/Standardized estimates	Mediation type observed
H^2^	Product Pricing → Satisfaction → Consumer Buying Behavior	0.23***	0.35	0.16	Full Mediation
H^4^	Product Packaging → Satisfaction → Consumer Buying Behavior	0.17**	0.29***	0.09	Partial Mediation

*** = Significance level at 1%.** = Significance level at 5%.

### Hypothetical Results

The results of the hypothesis are shown in Table 9 in a more detailed and comprehensive manner. To calculate the standard error with T and P-values and the significance of the path coefficient, bootstrapping (1,000 subsamples) was used, which provided direct evidence of the hypotheses being accepted or rejected. The structural model analysis results show the path coefficients and their significance levels, as presented in Table 9. The findings confirmed that all five relationships were significant, and it can be concluded that H_1_, H_2_, H_3_, H_4_, and H_5_ were supported.

**TABLE 9:** Hypothesis results.

S/R	Research question	Hypothesis		Results
1	RQ^1^: Is there a relationship between product pricing and buyer decision process?	Null	Product Pricing is not a significantly positive predictor of buyer decision process	Rejected
		Alternative	Product Pricing is a significantly positive predictor of buyer decision process	Accepted
2	RQ^2^: Is there a relationship between product packaging and buyer decision process?	Null	Product Packaging is not a significantly positive predictor of buyer decision process	Rejected
		Alternative	Product Packaging is a significantly positive predictor of buyer decision process	Accepted
3	RQ^3^: Is there a relationship between satisfaction and buyer decision process?	Null	Satisfaction is not a significantly positive predictor of buyer decision process	Rejected
		Alternative	Satisfaction is a significantly positive predictor of buyer decision process	Accepted
4	RQ^4^: Is there a mediating effect of satisfaction among product pricing and buyer decision process?	Null	There is no significantly positive mediating impact of satisfaction among product pricing and buyer decision process.	Rejected
		Alternative	There is a significantly positive mediating impact of satisfaction among product pricing and buyer decision process.	Accepted
5	RQ^5^: Is there a mediating effect of satisfaction among product packaging and buyer decision process?	Null	There is no significantly positive mediating impact of satisfaction among product packaging and buyer decision process.	Rejected
		Alternative	There is a significantly positive mediating impact of satisfaction among product packaging and buyer decision process.	Accepted

## Discussion

According to Sisodiya and Sharma (2018), the marketing mix has a significant influence on the buying behavior of consumers. In this study, the main principle in packaging is to “reach a greater height of opportunity.” It is often regarded as a critical component of purchase decision making, and has often been shown to be a way of building market awareness and connecting with consumers outside the product itself and across several channels (Rambabu and Porika, 2020; Sadiq W. et al., 2020). Packaging performs multidimensional functions. It can not only offer knowledge about products and business entities, but it is also a technique for communicating with consumers and safeguarding product quality (Silayoi and Speece, 2007). Pricing can be considered one of the most vital and essential elements that can influence consumer buying behavior or the buyer decision process (Dhurup et al., 2014; Sadiq W. et al., 2020).

According to Kotler et al. (2012), customer satisfaction “is the extent to which a product’s perceived performance matches the buyer’s expectations.” Aslam et al. (2018) stated that price has a positive and significant correlation with customer satisfaction. Furthermore, they believed that the success of the sector was based on price fairness and customer satisfaction. Previous studies have also discussed this phenomenon in connection with other geographical locations. The price factor is more relatable to consumer buying behavior than product packaging (Jabarzare and Rasti-Barzoki, 2020; Huo et al., 2021). Product pricing has a greater influence than product packaging on the buyers’ decision processes (Pratama and Suprapto, 2017; Abdullah et al., 2021). Innovation in product packaging also has a significant relationship with the consumer; however, if any organization wants to follow a strategy that is relevant to its product packaging, then there are several strategies for this kind of plan of action. Most consumers desire a range of product choices when purchasing, in terms of packaging. Thus, the marketer should place a premium on creative and exclusive packaging that is distinctive in terms of scale, instruction, convenience, product design, and form when compared to rivals in market segmentation (Rundh, 2009; Bollen and Pearl, 2013). Product packaging serves as an excellent networking medium for target customers, eventually increasing their knowledge levels. Packaging must convey pertinent details about the product and brand, including ingredient composition, intent, and consistency. In addition, packaging should provide all of this material in regional languages to demonstrate respect for consumers. Efficient packaging is critical not only for storing and protecting goods but also for generating interest in and action toward buying the commodity. Currently, eco-friendly packaging is essential. Thus, advertisers should prioritize this factor and employ best practices to the maximum degree possible, including eco-friendly recyclable packaging (Deliza and MacFie, 2001; Abdullah et al., 2021).

## Conclusion and Recommendations

The study results clearly show that both product pricing and packaging have a statistically significant relationship with the buyer’s decision process. At the same time, the introduction of satisfaction leads to the observation of full mediation in the case of product pricing and partial mediation in product packaging. Despite knowing that both the variables have a statistically significant relationship with the consumer buying behavior, it is essential to understand the managerial implications. Suppose, we would like to report and recommend these findings to different organizations looking to cut their operational costs in any possible way without compromising product quality, we suggest in such cases that they focus on pricing strategies for a better consumer response. A focus on the product packaging design process, packaging material, or the information available on product packaging positively influences consumer buying behavior. However, its effect is lower than product pricing. Therefore, it is recommended for managers that if they want to connect with their target customers more efficiently and effectively, they should focus on both product pricing and packaging options. However, if they can afford only one option from the product’s operational cost perspective, they must focus on product pricing strategies.

In future studies, it must be kept in mind that these findings pertain directly to the individuals listed as respondents. To make it more accurate, other demographic, psychographic, and geographic samples should be used. It is likely that when data are thus obtained, the findings will differ. To ensure more lasting and repeatable corporate outcomes, several studies are required to obtain results that are more accurate and reliable.

## Data Availability Statement

The original contributions presented in the study are included in the article/supplementary material, further inquiries can be directed to the corresponding author.

## Author Contributions

HZ, XY, and ZL contributed to conception and design of the study. HZ organized the database, performed the statistical analysis, and wrote the first draft of the manuscript. XY, ZL, and QY wrote sections of the manuscript. All authors contributed to manuscript revision, read, and approved the submitted version.

## Conflict of Interest

The authors declare that the research was conducted in the absence of any commercial or financial relationships that could be construed as a potential conflict of interest.

## Publisher’s Note

All claims expressed in this article are solely those of the authors and do not necessarily represent those of their affiliated organizations, or those of the publisher, the editors and the reviewers. Any product that may be evaluated in this article, or claim that may be made by its manufacturer, is not guaranteed or endorsed by the publisher.

## Abbreviations

CFA: Confirmatory Factor Analysis
RMSEA: Root Mean Square of Error Approximation
CFI: Comparative fit index
EDT: Expectancy Disconfirmation Theory
SCT: Social Cognitive Theory.
